# Association of short-term exposure to fine particulate air pollution and mortality: effect modification by oxidant gases

**DOI:** 10.1038/s41598-018-34599-x

**Published:** 2018-10-31

**Authors:** Eric Lavigne, Richard T. Burnett, Scott Weichenthal

**Affiliations:** 1 Air Health Science Division, Health Canada, Ottawa, ON Canada; 2 School of Epidemiology & Public Health, University of Ottawa, Ottawa, Ontario Canada; 3 Population Studies Division, Health Canada, Ottawa, ON Canada; 4 Department of Epidemiology, Biostatistics, and Occupational Health, McGill University, Montreal, QC Canada

## Abstract

Short term changes in exposure to outdoor fine particulate matter (PM_2.5_) concentrations are associated with an increased risk of mortality. However, less is known about how oxidant gases may modify the acute health effects of PM_2.5_. Our objective was to investigate whether associations between acute exposure to PM_2.5_ and mortality were modified by the oxidant gases O_3_ and NO_2_ using their redox-weighted average (O_x_). We conducted a multi-city case-crossover study in 24 cities across Canada between 1998–2011 including 1,179,491 nonaccidental mortality events. Interquartile increases in lag-0 and 3-day mean PM_2.5_ and O_x_ concentrations were each associated with small increases in nonaccidental and cardiovascular mortality. In stratified analyses, associations between PM_2.5_ and nonaccidental and cardiovascular mortality tended to be greatest in the highest tertile of O_x_ with a significant interaction observed between lag 0 PM_2.5_ and 3-day mean O_x_ (interaction p-value = 0.04). There was no evidence of effect modification by O_x_ in the relationship between PM_2.5_ and respiratory mortality. Overall, the relationship between short-term changes in outdoor PM_2.5_ and nonaccidental mortality may be greater when oxidant gas concentrations are also elevated. In some regions, reductions in oxidant gas concentrations may also reduce the acute health impacts of PM_2.5_.

## Introduction

Short-term increases in outdoor fine particulate air pollution (PM_2.5_) are known to be associated with increased mortality^1–3^. Other pollutants including nitrogen dioxide (NO_2_) and ozone (O_3_) have also been associated with daily mortality events^4,5^, but it is not clear how these oxidant gases may modify the acute health effects of PM_2.5._ This is an important public health issue as populations are simultaneously exposed to both PM_2.5_ and oxidant gases (e.g. O_3_ and NO_2_). Moreover, understanding interactions between these pollutants may help to inform preventative measures aimed at reducing the public health impacts of outdoor air pollution.

A recent study conducted in London, England found that O_3_, NO_2_, and their combined oxidant capacity (O_x_) were each associated with daily mortality with the strongest associations observed for O_x_^6^. However, this study did not specifically evaluate how O_x_ may modify the acute health effects of PM_2.5_. Recently, we reported that the strength of associations between long-term exposures to outdoor PM_2.5_ and nonaccidental, cardiovascular, and respiratory mortality were greater in regions with higher O_x_ concentrations^7^. Biological mechanisms explaining this observation may include the fact that oxidant gases are known to deplete anti-oxidants in the lung lining fluid^8^ and increase the permeability of the lung epithelium^9–12^. Alternatively, photochemical aging of PM_2.5_ may increase particle toxicity^13,14^ and this process may be accelerated in regions with higher oxidant gas concentrations. In either case, this evidence suggests that PM_2.5_ may be more harmful on days with increased concentrations of oxidant gases.

In this study, we examined how O_x_ may modify the acute health effects of PM_2.5_ using a multi-city case-crossover study of non-accidental, cardiovascular, and respiratory mortality.

## Results

Descriptive statistics are provided in Tables 1 and 2. In total, 1,179,491 nonaccidental mortality events occurred, including 401,719 cases of cardiovascular mortality and 105,980 cases of respiratory mortality. Nonaccidental mortality cases tended to be younger than cases of cardiovascular or respiratory deaths and both genders were present in approximately equal proportions. Table 2 shows the distribution of ambient air pollutants and weather variables in 24 cities across Canada during the study period. Mean daily concentrations were 8.84 µg/m^3^ for PM_2.5_, 16.48 ppb for NO_2_, 20.87 ppb for O_3_ and 19.38 for O_x_. The average daily mean temperature was 7.36 °C, varying from −39.7 to 31.51 °C (interquartile range of 15.3 °C). Table S1 shows Pearson correlation coefficients among the air pollutants and weather variables. PM_2.5_ was moderately correlated with NO_2_ and weakly correlated with the other pollutants and weather variables.

**Table 1:** Distribution of the number of deaths for nonaccidental, cardiovascular, and respiratory mortality in 24 cities across Canada (1998–2011).

Outcome	Number of deaths	% Male	Mean Age (years)
Nonaccidental deaths	1,179,491	49.7	75.0
All cardiovascular deaths	401,719	49.5	79.0
All respiratory deaths	105,980	50.5	80.1

**Table 2:** Daily concentrations of ambient air pollutants and weather variables in 24 cities across Canada (1998–2011).

Air Pollutants & weather variables	Mean (SD)	Median	IQR	Range
PM_2.5_ (µg/m^3^)	8.84 (6.50)	7.06	6.63	<1-98.15
NO_2_ (ppb)	16.48 (8.33)	15.36	10.91	<1-68.44
O_3_ (ppb)	20.87 (9.99)	20.12	13.61	<1-89.78
O_x_ (ppb)	19.38 (6.25)	18.73	8.13	<1-62.40
Temperature (°C)	7.36 (10.62)	8.00	15.3	−39.7–31.51
Relative Humidity (%)	72.22 (12.85)	72.96	17.41	16.04–100

IQR, interquartile range; O_x_, redox-weighted oxidant capacity of NO_2_ and O_3._

**Figure 1:**
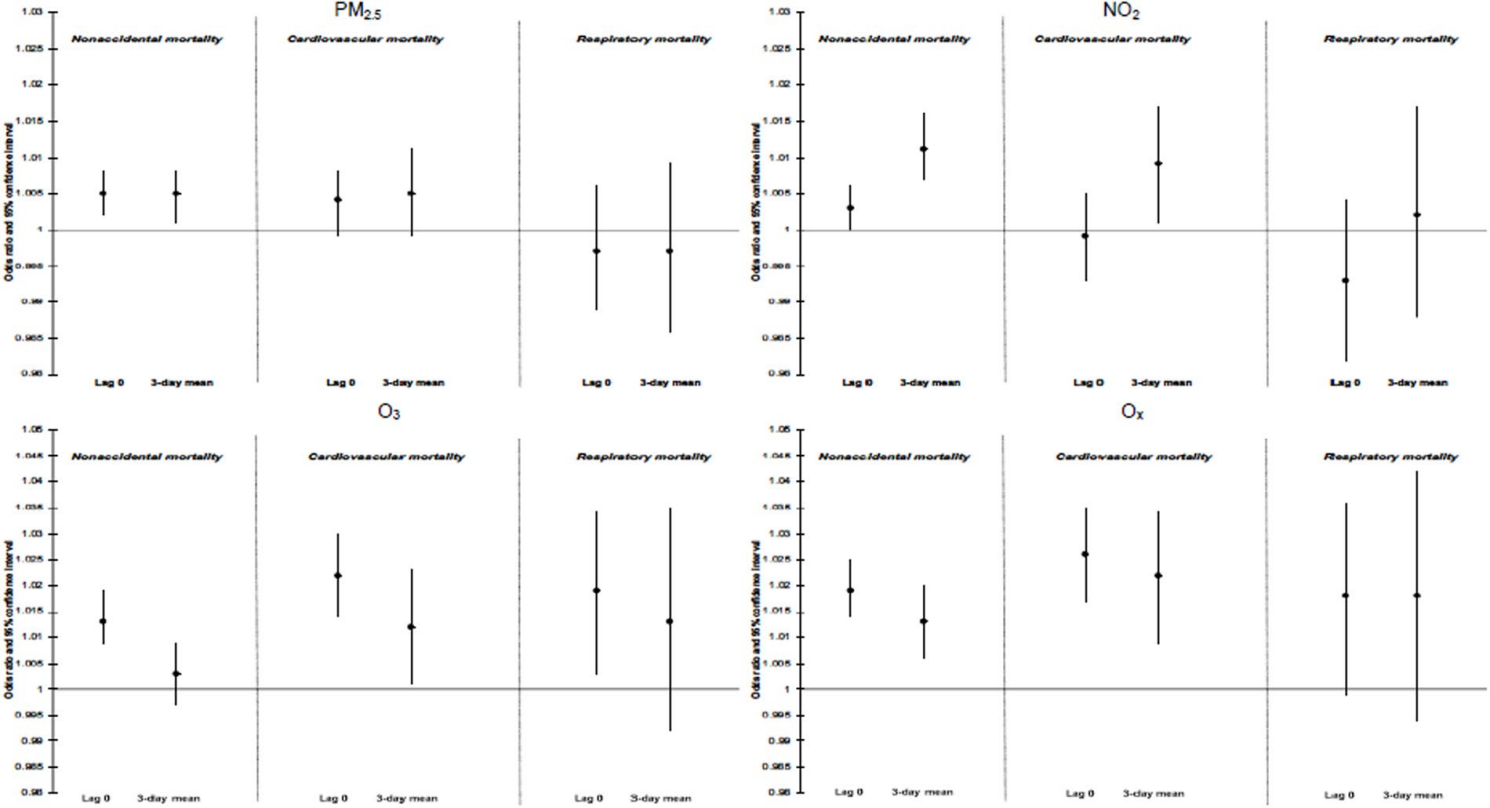
Odds ratios (ORs)^1^ and 95% CIs for nonaccidental, cardiovascular, and respiratory mortality associated with acute exposure to ambient air pollutants in 24 cities across Canada (1998–2011). ORs reflect a 6.63 µg/m^3^ change in PM_2.5_, a 10.91 ppb change in NO_2_, a 13.61 ppb change in O_3_, and a 8.13 ppb change in O_x_. All models are adjusted for 3-day mean ambient temperature (cubic splines) and relative humidity and daily counts of hospitalization for influenza (in respiratory mortality models only).

Figure 1 and Table S2 show associations between ambient air pollutants and nonaccidental, cardiovascular and respiratory mortality during the time period of 1998 to 2011. For PM_2.5_, lag-0 and 3-day mean concentrations were each associated with small increases in nonaccidental and cardiovascular mortality. Short term changes in lag-0 and 3-day mean O_x_ concentrations were positively associated with all three mortality outcomes, but 95% confidence intervals for respiratory mortality included the null. In general, risk estimates for O_x_ tended to be slightly larger than for O_3_ or NO_2_ individually with the exception of lag-0 respiratory mortality which was similar for O_3_ and O_x_.

In single pollutant models, the strongest association was between lag-0 O_x_ and cardiovascular mortality (OR = 1.026; 95% CI: 1.017, 1.035 per 10.91 ppb). As sensitivity analyses, we examined two-pollutant models including linear terms for both PM_2.5_ and O_x_. In these models, O_x_ remained positively associated with nonaccidental (OR = 1.011, 95% CI: 1.004, 1.018) and cardiovascular mortality (OR = 1.020, 95% CI: 1.008, 1.033) whereas risk estimates for PM_2.5_ decreased slightly (nonaccidental: OR = 1.005, 95% CI: 1.001, 1.008; cardiovascular: OR = 1.005, 95% CI: 0.999, 1.011) (Tables S3 and S4).

**Figure 2:**
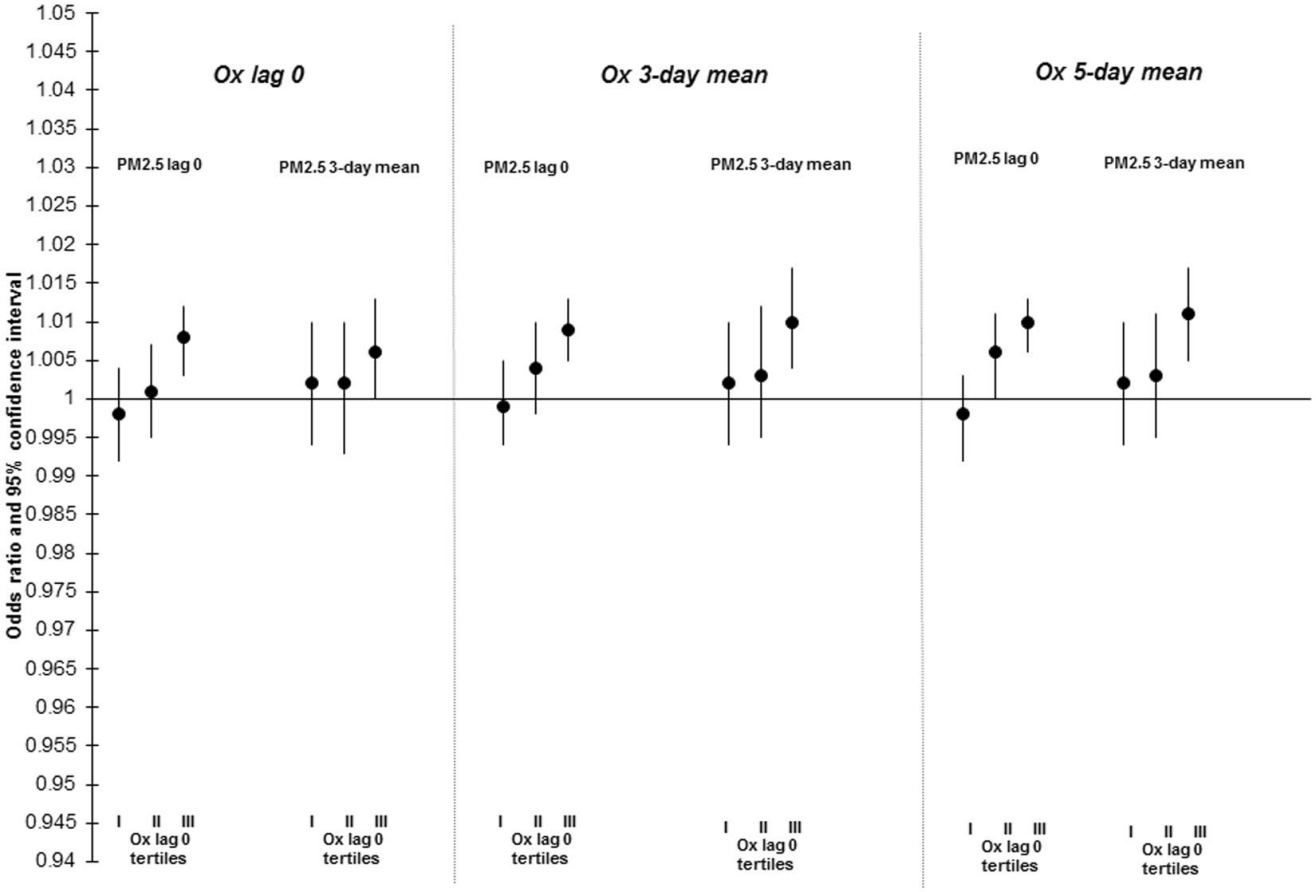
Odds ratios (ORs)^1^ and 95% CIs for associations between lag 0 and 3-day mean PM_2.5_ and nonaccidental mortality across tertiles (I, II, III) of same day, 3-day mean, and 5-day mean O_x_ in 24 cities across Canada (1998–2011). ORs reflect a 6.63 µg/m^3^ change in PM_2.5_. All models are adjusted for 3-day mean ambient temperature (cubic splines) and relative humidity.

**Figure 3:**
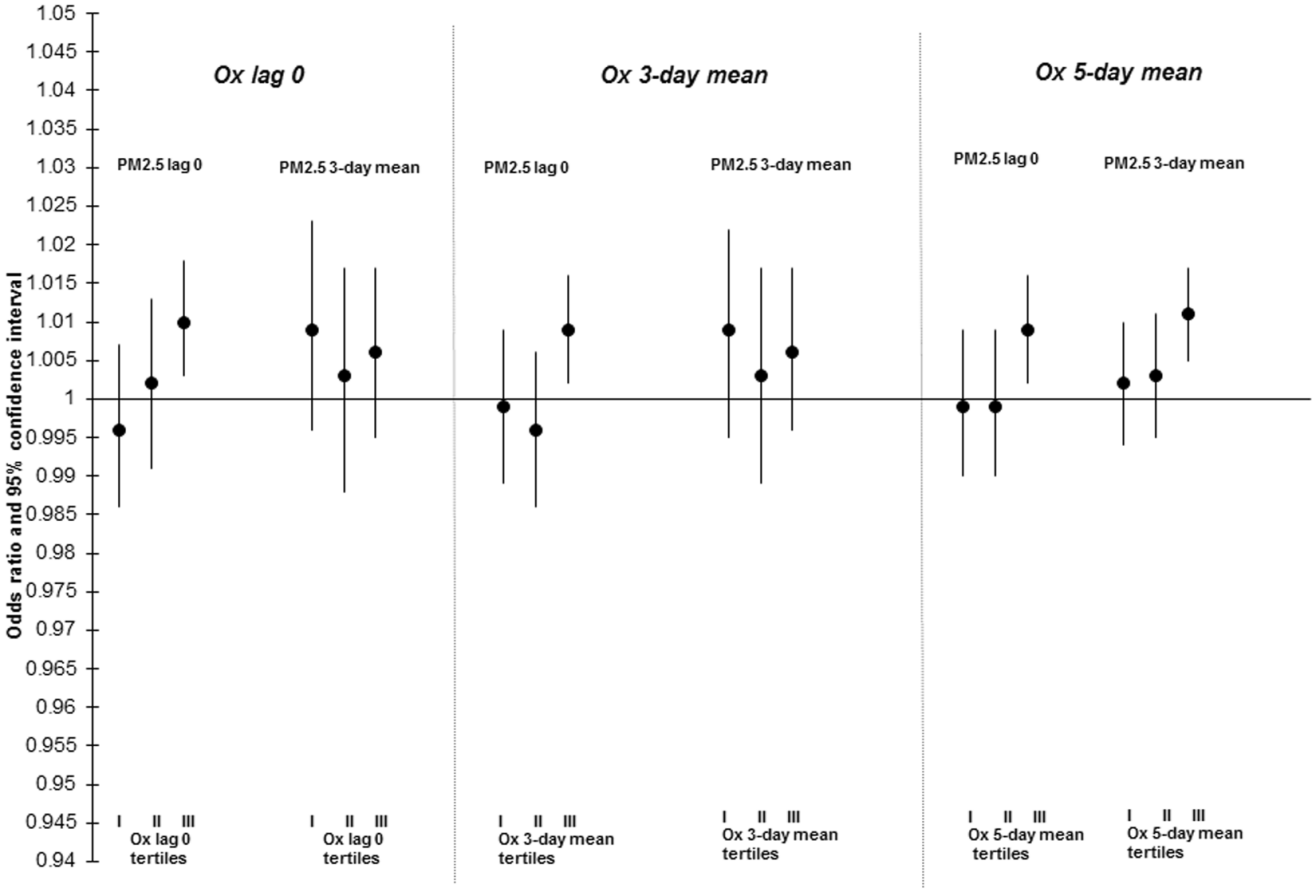
Odds ratios (ORs)^1^ and 95% CIs for associations between lag 0 and 3-day mean PM_2.5_ and cardiovascular mortality across tertiles (I, II, III) of same day, 3-day mean, and 5-day mean O_x_ in 24 cities across Canada (1998–2011). ORs reflect a 6.63 µg/m^3^ change in PM_2.5_. All models are adjusted for 3-day mean ambient temperature (cubic splines) and relative humidity.

**Figure 4:**
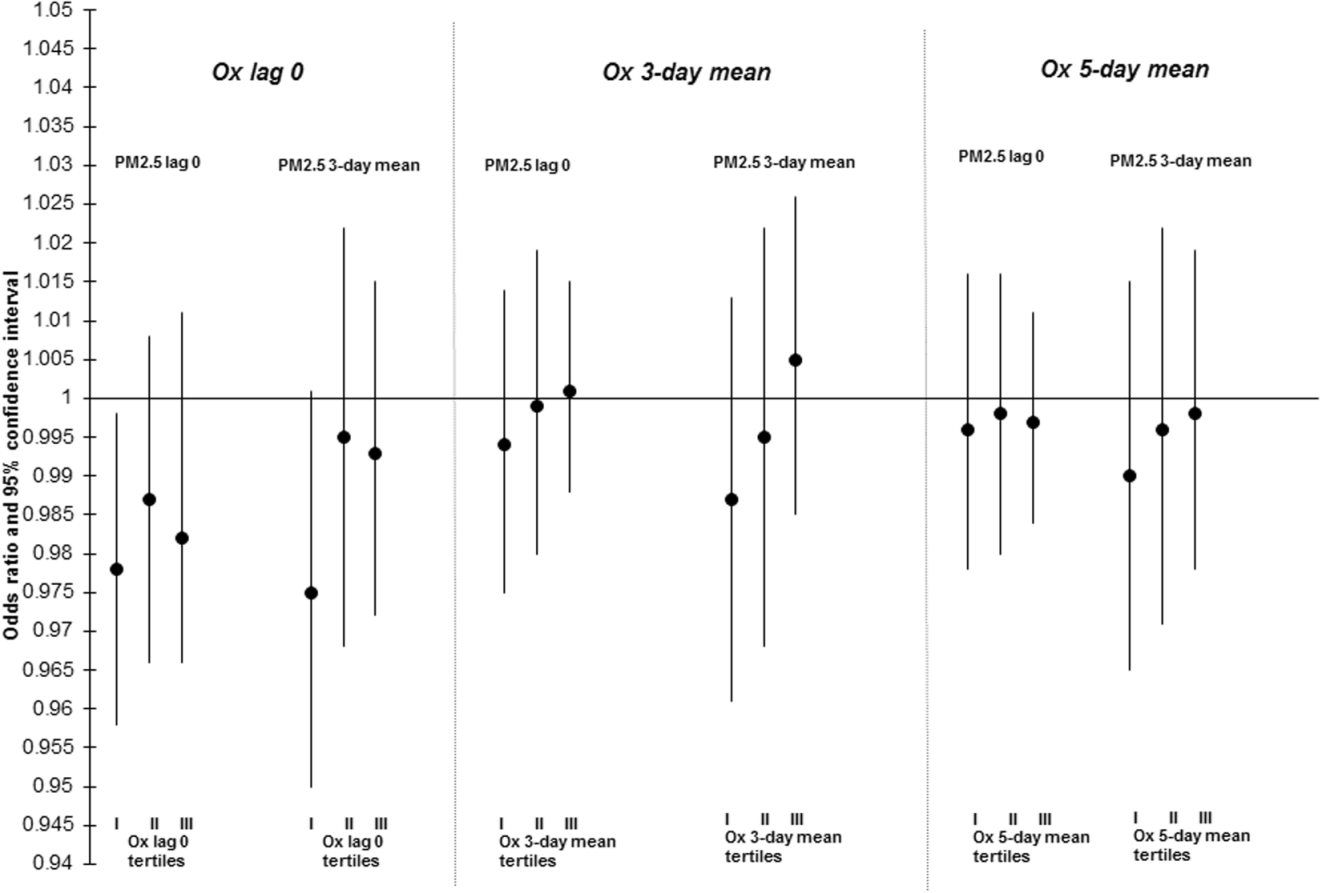
Odds ratios (ORs)^1^ and 95% CIs for associations between lag 0 and 3-day mean PM_2.5_ and respiratory mortality across tertiles (I, II, III) of same day, 3-day mean, and 5-day mean O_x_ in 24 cities across Canada (1998–2011). ORs reflect a 6.63 µg/m^3^ change in PM_2.5_. All models are adjusted for 3-day mean ambient temperature (cubic splines) and relative humidity and daily counts of hospitalization for influenza.

The results of stratified analyses examining the relationship between PM_2.5_ and mortality across tertiles of O_x_ are presented in Figs 2–4 and Table S5. For lag-0 PM_2.5_, increased risks of nonaccidental (interaction p-value = 0.04) and cardiovascular mortality (interaction p-value = 0.19) were limited to the highest tertiles of O_x_. This trend was less clear for 3-day PM_2.5_ concentrations and evidence of effect modification by O_x_ was not observed for the relationship between PM_2.5_ and respiratory mortality. In sensitivity analyses, PM_2.5_-mortality associations were not modified by NO_2_ or O_3_ individually (data not shown). As well, findings when restricted to the warm season only were similar to the whole year analyses (data not shown).

## Discussion

In this study, we examined how oxidant gases may modify associations between short-term changes in outdoor PM_2.5_ concentrations and nonaccidental, cardiovascular, and respiratory mortality. As in previous studies, we found that short-term changes in ambient air pollution concentrations were associated with small increased risks of mortality, predominantly nonaccidental and cardiovascular mortality. For PM_2.5_ specifically, we noted that same day exposures were only associated with nonaccidental and cardiovascular mortality during periods with the highest O_x_ concentrations (i.e. above 21.38 ppb). Moreover, we found that short term changes in O_x_ were more strongly associated with nonaccidental and cardiovascular mortality than PM_2.5_ in mutually adjusted models.

One previous study conducted in London, England reported an association between daily variations in O_x_ and mortality^6^, but to our knowledge this is the first study to evaluate how oxidant gases may modify the relationship between short-term changes in outdoor PM_2.5_ and mortality. However, we recently reported that O_x_ levels modified the relationship between short-term changes in ambient PM_2.5_ and the risk of myocardial infarction and our results for cardiovascular mortality are consistent with this finding^15^. In addition, we previously reported that oxidant gases modified the relationship between long-term PM_2.5_ exposures and mortality with stronger associations observed in areas with higher O_x_ concentrations^7^. Collectively, these findings suggest that oxidant gases may modify both the acute and chronic health effects of PM_2.5_ exposures with larger risk estimates observed for chronic health impacts.

While our study could not directly evaluate how O_x_ concentrations may modify PM_2.5_ health impacts existing evidence suggests that such a relationship is biologically plausible. For example, one possibility is that oxidant gases deplete anti-oxidants in the lung lining fluid which in turn may lower our natural defense against reactive oxygen species generated in response to PM_2.5_^8,16^. Moreover, some findings suggest that the lung epithelium barrier is more permeable following ozone exposures and this may facilitate the absorption of particles and/or inflammatory mediators from the lungs directly into the systemic circulation^9–12^. On the other hand, increased O_x_ concentrations may influence the toxicity of particles themselves as photochemical aging has been shown to increase particle toxicity^13,14^.

While this study had a number of important strengths including a large number of mortality cases from multiple cities across Canada it is important to recognize several limitations. First, as in all epidemiological studies, exposure measurement error likely impacted our results as mean daily air pollution concentrations were assigned to case and control periods using fixed-site monitors at the city level. This error was likely most important for NO_2_ exposures as within-city spatial variations are greater for NO_2_ than for O_3_ or PM_2.5_ and fixed-site measurements may not adequately represent spatial differences in NO_2_ exposures over large geographic areas. However, assuming that measurement errors are non-differential between case and control periods, this would usually result in an underestimation of risk estimates and is not a likely explanation of increased PM_2.5_ mortality association in upper tertiles of O_x_. Satellite based air pollutants modeling have been proved as an effective method to accurately capture the spatial variability of ambient air pollution^17–19^. However, in this study, we could not obtain daily satellite-based air pollutant concentrations, as these are mainly available on a long-term basis across Canada^20^. In addition, we relied on data from 1998 to 2011 and thus more recent years are excluded from our analyses.

In summary, our results suggest that oxidant gases may act to strengthen associations between same day PM_2.5_ exposures and nonaccidental and cardiovascular mortality. While these risks remain small, they suggest that the health benefits of reductions in O_x_ concentrations may be larger than expected as such reductions may also decrease the health impacts of PM_2.5_ even if mass concentrations remain unchanged.

## Methods

### Study population

A time-stratified case crossover study design^21^ was used to estimate associations between short-term changes in outdoor air pollution concentrations and the risk of non-accidental (ICD-10: A00 to R99), cardiovascular (ICD-10: I10–99), and respiratory mortality (ICD-10: J00-J99). Mortality data were obtained for the years 1998 to 2011 from the Canadian Mortality Database maintained by Statistics Canada. All subjects who died and were residents of the corresponding cities under investigation were eligible to be included in the analyses. The following 24 cities across Canada were included: Abbotsford, Calgary, Edmonton, Halifax, Hamilton, Kingston, Kitchener, London, Montreal, Oakville, Oshawa, Ottawa, Regina, Saint John (New Brunswick), Sarnia, Saskatoon, Sault Ste Marie, St-John’s (New Found Land and Labrador), Thunder Bay, Toronto, Vancouver, Victoria, Windsor, Winnipeg.

### Daily Air Pollution Data

Daily average concentrations of ambient PM_2.5_, NO_2_ and O_3_ were obtained from fixed-site monitoring stations operated by the National Air Pollution Surveillance (NAPS) network maintained by Environment Canada. Daily mean temperature and relative humidity data were also collected from weather stations in the corresponding cities. If daily air pollution concentrations were available for multiple monitors in a single city, daily concentrations were averaged over all available monitors. We calculated the combined oxidant capacity (O_x_) of O_3_ and NO_2_ for each day in each city using a weighted average with weights equivalent to their respective redox potentials (i.e. O_x_ = [(1.07 × NO_2_) + (2.075 × O_3_)]/3.145)^22^. Exposures were assigned to case and control periods based on the monitoring station located in each subjects’ city of residence.

### Statistical analysis

Conditional logistic regression models were used to estimate the association between short-term changes in ambient air pollutant concentrations and the risk of mortality^21^. All models pooled cases across cities using a random intercept at the city level to account for potential within-city correlations. We developed models for the whole year and separately for the warm season (April–September) in order to specifically capture the portion of the year with elevated O_3_ concentrations_._ All ambient air pollutants (i.e. PM_2.5_, NO_2_, O_3_, and O_x_, as defined above) were evaluated in single pollutant models and all odds ratios (and 95% confidence intervals (CI)) reflect interquartile range (IQR) changes in pollutant concentrations. We used lag-0 IQR values for all statistical analyses since interquartile ranges were similar across exposure lag periods (within 1 µg/m^3^ for PM_2.5_ and within 1 ppb for NO_2_ and O_3_).

We evaluated two different exposure periods for ambient air pollutants: lag-0 (the same day as the mortality event or the control period) and 3-day mean concentrations (including the day of the mortality even or the control period). As sensitivity analyses we also examined the time periods lag-1 (the day prior to the event or the control period) and lag-2 (two days prior to the event or the control period); the magnitudes of these associations were similar to or less than values for the main analyses and are not discussed further. Since the case-crossover design compares cases to themselves at different points in time it adjusts for factors that do not vary within individuals over short time-periods (e.g. age, smoking status, body mass index). In this study, the case period consisted of the day of the mortality event and control periods were selected on the same day of the week in the same month and year as the case period. This time-stratified approach to referent selection has been shown to result in unbiased conditional logistic regression estimates in case-crossover studies^23^. All models were adjusted for 3-day mean ambient temperature with a quadratic B-spline with three internal knots placed at the 10th, 75th, and 90th percentiles of location-specific temperature distributions and 3-day mean relative humidity^24^.

To evaluate effect modification by O_x_ in the relationship between acute exposure to PM_2.5_ and mortality we conducted stratified analyses across tertiles of O_x_ (<16.41 ppb, 16.41–<21.38 ppb, ≥21.38 ppb) based on the distribution of O_x_ across all cities. We evaluated the statistical significance of effect modification by including a cross-product interaction term between PM_2.5_ and the categorical variable for tertiles of O_x_. Wald’s method was used to assess the presence of interaction on the multiplicative scale. Effect modification was considered statistically significant if the p-value for the interaction term was less than 0.05.

As sensitivity analyses, we investigated two-pollutant models to evaluate the extent to which PM_2.5_-mortality associations may be confounded by O_x_. We also evaluated effect modification of PM_2.5_-mortality associations across tertiles of NO_2_ and O_3_. Finally, we investigated effect modification of O_x_ in the warm season only. All statistical analyses were conducted with R software (version 3.2.4) using the packages *dlnm* and *lme4*.

### Institutional Approvals

The use of the data in this study was approved by the Statistics Canada Policy Committee after consultation with the Statistics Canada Confidentiality and Legislation Committee, Data Access and Control Services Division, and the Federal Privacy Commissioner. This approval is equivalent to that of standard research ethics boards.

## Electronic supplementary material

Supplemental material
