# Supplementary material for: Association of short-term exposure to fine particulate air pollution and mortality: effect modification by oxidant gases
Source: Sci Rep. 2018 Oct 31;8:16097. doi: 10.1038/s41598-018-34599-x (PMC6208424; doi:10.1038/s41598-018-34599-x)
Supplement: Supplementary file 1 — Supplemental material [file 41598_2018_34599_MOESM1_ESM.docx]

**Supplemental material**

**Association of short-term exposure to fine particulate air pollution and mortality: effect modification by oxidant gases**

Eric Lavigne^1,2^, Richard T Burnett^3^, Scott Weichenthal^1,4^

^1^Air Health Science Division, Health Canada, Ottawa, ON, Canada

^2^ School of Epidemiology & Public Health, University of Ottawa, Ottawa, Ontario, Canada

^3^Population Studies Division, Health Canada, Ottawa, ON, Canada

^4^ Department of Epidemiology, Biostatistics, and Occupational Health, McGill University, Montreal, QC, Canada

**Table S1**. Pearson correlation coefficients of mean concentrations of ambient air pollutants and weather variables in 24 cities across Canada (1998 - 2011).

|  | PM_2.5_ (µg/m^3^) | NO_2_ (ppb) | O_3_ (ppb) | O_x_ (ppb) | Temperature (^o^C) | Relative Humidity (%) |
| --- | --- | --- | --- | --- | --- | --- |
| PM_2.5_ (µg/m^3^) | 1 | 0.53 | 0.03 | 0.28 | 0.08 | 0.04 |
| NO_2_ (ppb) |  | 1 | -0.33 | 0.10 | -0.24 | -0.07 |
| O_3_ (ppb) |  |  | 1 | 0.90 | 0.32 | -0.39 |
| O_x_ (ppb) |  |  |  | 1 | 0.23 | -0.44 |
| Temperature (^o^C) |  |  |  |  | 1 | -0.12 |
| Relative Humidity (%) |  |  |  |  |  | 1 |

**Table S2**. Odds ratios (ORs) and 95% CIs for nonaccidental, cardiovascular, and respiratory mortality associated with acute exposure to ambient air pollutants in 24 cities across Canada (1998 - 2011).

| Cause of death | Lag | Exposure | | | |
| --- | --- | --- | --- | --- | --- |
|  |  | PM_2.5_ | NO_2_ | O_3_ | O_x_ |
|  |  | OR (95% CI) | OR (95% CI) | OR (95% CI) | OR (95% CI) |
| Nonaccidental | 0 | 1.005 (1.002 – 1.008) | 1.003 (1.000 – 1.006) | 1.013 (1.009 – 1.019) | 1.019 (1.014 – 1.025) |
|  | 1 | 1.006 (1.004 – 1.009) | 1.012 (1.009 – 1.016) | 0.997 (0.992 – 1.002) | 1.006 (1.001 – 1.011) |
|  | 2 | 0.997 (0.994 – 0.999) | 1.004 (1.001 – 1.007) | 0.994 (0.990 – 0.999) | 0.996 (0.991 – 1.001) |
|  | 3-day average | 1.005 (1.001 – 1.008) | 1.011 (1.007 – 1.016) | 1.003 (0.997 – 1.009) | 1.013 (1.006 – 1.020) |
|  |  |  |  |  |  |
| Cardiovascular | 0 | 1.004 (0.999 – 1.008) | 0.999 (0.993 – 1.005) | 1.022 (1.014 – 1.030) | 1.026 (1.017 – 1.035) |
|  | 1 | 1.008 (1.003 – 1.012) | 1.013 (1.007 – 1.019) | 1.001 (0.993 – 1.009) | 1.012 (1.003 – 1.020) |
|  | 2 | 0.997 (0.993 – 1.002) | 1.004 (0.998 – 1.009) | 0.996 (0.989 – 1.004) | 0.998 (0.989 – 1.007) |
|  | 3-day average | 1.005 (0.999 – 1.011) | 1.009 (1.001 – 1.017) | 1.012 (1.001 – 1.023) | 1.022 (1.009 – 1.034) |
|  |  |  |  |  |  |
| Respiratory | 0 | 0.997 (0.989 – 1.006) | 0.993 (0.982 – 1.004) | 1.019 (1.003 – 1.034) | 1.018 (0.999 – 1.036) |
|  | 1 | 1.000 (0.991 – 1.009) | 1.009 (0.998 – 1.020) | 1.000 (0.985 – 1.015) | 1.007 (0.990 – 1.025) |
|  | 2 | 0.999 (0.990 – 1.007) | 1.002 (0.991 – 1.013) | 1.003 (0.989 – 1.018) | 1.006 (0.989 – 1.023) |
|  | 3-day average | 0.997 (0.986 – 1.009) | 1.002 (0.988 – 1.017) | 1.013 (0.992 – 1.035) | 1.018 (0.994 – 1.042) |

ORs reflect a 6.63 µg/m^3^ change in PM_2.5_, a 10.91 ppb change in NO_2_, a 13.61 ppb change in O_3_, and a 8.13 ppb change in O_x_. All models are adjusted for 3-day mean ambient temperature (cubic splines) and relative humidity and daily counts of hospitalization for influenza (in respiratory mortality models only).

**Table S3**. Potentiation of the relationship between lag 0 exposure to ambient PM_2.5_ and mortality (nonaccidental, cardiovascular and respiratory mortality) by 3-day mean O_x_ in 24 cities across Canada (1998 - 2011).

| Cause of Death | AIC | Pollutant | | | | | |
| --- | --- | --- | --- | --- | --- | --- | --- |
|  |  | PM_2.5_ | | O_x_ | | PM_2.5_*O_x_ | |
|  |  | OR | 95% CI | OR | 95% CI | OR | 95% CI |
| ***PM_2.5_*** |  |  |  |  |  |  |  |
| Nonaccidental | 3459084 | 1.005 | 1.002 – 1.008 |  |  |  |  |
| Cardiovascular | 1177947 | 1.004 | 0.999 – 1.009 |  |  |  |  |
| Respiratory | 310592 | 0.997 | 0.989 – 1.009 |  |  |  |  |
| ***PM_2.5_ + O_x_*** | |  |  |  |  |  |  |
| Nonaccidental | 3459078 | 1.005 | 1.002 – 1.007 | 1.010 | 1.003 – 1.017 |  |  |
| Cardiovascular | 1177939 | 1.002 | 0.998 – 1.007 | 1.020 | 1.008 – 1.033 |  |  |
| Respiratory | 310592 | 0.996 | 0.988 – 1.005 | 1.020 | 0.995 – 1.044 |  |  |
| ***PM_2.5 +_ O_x +_ PM_2.5_*O_x_*** |  |  |  |  |  |  |  |
| Nonaccidental | 3459080 | 1.002 | 0.995 – 1.009 | 1.008 | 0.999 – 1.017 | 1.002 | 0.997 – 1.006 |
| Cardiovascular | 1177940 | 1.000 | 0.988 – 1.012 | 1.018 | 1.002 – 1.034 | 1.002 | 0.994 – 1.010 |
| Respiratory | 310593 | 1.001 | 0.977 – 1.026 | 1.024 | 0.993 – 1.056 | 0.997 | 0.981 – 1.012 |

ORs reflect a 6.63 µg/m^3^ change in PM_2.5_, a 8.13 ppb change in O_x_ and 100 ppb*µg/m^3^ change in PM_2.5_*O_x._ All models are adjusted for 3-day mean ambient temperature (cubic splines) and relative humidity and daily counts of hospitalization for influenza (in respiratory mortality models only).

**Table S4**. Potentiation of the relationship between 3-day mean ambient PM_2.5_ and mortality (nonaccidental, cardiovascular and respiratory mortality) by 3-day mean O_x_ in 24 cities across Canada (1998 - 2011).

| Cause of Death | AIC | Pollutant | | | | | |
| --- | --- | --- | --- | --- | --- | --- | --- |
|  |  | PM_2.5_ | | O_x_ | | PM_2.5_*O_x_ | |
|  |  | OR | 95% CI | OR | 95% CI | OR | 95% CI |
| ***PM_2.5_*** |  |  |  |  |  |  |  |
| Nonaccidental | 3459094 | 1.005 | 1.001 – 1.008 |  |  |  |  |
| Cardiovascular | 1177947 | 1.005 | 0.999 – 1.011 |  |  |  |  |
| Respiratory | 310592 | 0.997 | 0.986 – 1.009 |  |  |  |  |
| ***PM_2.5_ + O_x_*** | |  |  |  |  |  |  |
| Nonaccidental | 3459087 | 1.004 | 1.000 – 1.007 | 1.011 | 1.004 – 1.018 |  |  |
| Cardiovascular | 1177939 | 1.003 | 0.997 – 1.009 | 1.020 | 1.008 – 1.033 |  |  |
| Respiratory | 310592 | 0.996 | 0.984 – 1.007 | 1.019 | 0.995 – 1.044 |  |  |
| ***PM_2.5 +_ O_x +_ PM_2.5_*O_x_*** |  |  |  |  |  |  |  |
| Nonaccidental | 3459088 | 1.008 | 1.000 – 1.017 | 1.015 | 1.005 – 1.025 | 0.997 | 0.992 – 1.002 |
| Cardiovascular | 1177938 | 1.014 | 0.999 – 1.029 | 1.030 | 1.013 – 1.048 | 0.992 | 0.983 – 1.002 |
| Respiratory | 310593 | 1.008 | 0.979 – 1.037 | 1.030 | 0.996 – 1.066 | 0.992 | 0.973 – 1.010 |

ORs reflect a 6.63 µg/m^3^ change in PM_2.5_, a 8.13 ppb change in O_x_ and 100 ppb*µg/m^3^ change in PM_2.5_*O_x._ All models are adjusted for 3-day mean ambient temperature (cubic splines) and relative humidity and daily counts of hospitalization for influenza (in respiratory mortality models only).

**Table S5**. Odds ratios (ORs)^1^ and 95% CIs for nonaccidental, cardiovascular, and respiratory mortality across tertiles (I, II, III) of same day, 3-day mean, and 5-day mean O_x_ in 24 cities across Canada (1998 - 2011).

| Cause of death | PM_2.5_ Lag | Time-Period of O_x_ Exposure | | | | | | | | | | | |
| --- | --- | --- | --- | --- | --- | --- | --- | --- | --- | --- | --- | --- | --- |
|  |  | Lag0 | | | | 3 Day Mean | | | | 5 Day Mean | | | |
|  |  | I | II | III | p value | I | II | III | p value | I | II | III | P value |
| Nonaccidental | 0 | 0.998  (0.992 – 1.004) | 1.001  (0.995 –  1.007) | 1.008  (1.003 – 1.012) | 0.05 | 0.999  (0.994 – 1.005) | 1.004  (0.998 – 1.010) | 1.009  (1.005 – 1.013) | 0.04 | 0.998  (0.992 – 1.003) | 1.006  (1.000 – 1.011) | 1.010  (1.006 – 1.013) | 0.01 |
|  | 3-day | 1.002  (0.994 – 1.010) | 1.002  (0.993 –  1.010) | 1.006  (1.000 – 1.013) | 0.35 | 1.002  (0.994 – 1.010) | 1.003  (0.995 – 1.012) | 1.010  (1.004 – 1.017) | 0.59 | 1.002  (0.994 – 1.010) | 1.003  (0.995 – 1.011) | 1.011  (1.005 – 1.017) | 0.36 |
|  |  |  |  |  |  |  |  |  |  |  |  |  |  |
| Cardiovascular | 0 | 0.996  (0.986 – 1.007) | 1.002  (0.991 –  1.013) | 1.010  (1.003 – 1.018) | 0.12 | 0.999  (0.989 – 1.009) | 0.996  (0.986 – 1.006) | 1.009  (1.002 – 1.016) | 0.19 | 0.999  (0.990 – 1.009) | 0.999  (0.990 – 1.009) | 1.009  (1.002 – 1.016) | 0.29 |
|  | 3-day | 1.009  (0.996 – 1.023) | 1.003  (0.988 –  1.017) | 1.006  (0.995 – 1.017) | 0.72 | 1.009  (0.995 – 1.022) | 1.003  (0.989 – 1.017) | 1.006  (0.996 – 1.017) | 0.38 | 1.002  (0.994 – 1.010) | 1.003  (0.995 – 1.011) | 1.011  (1.005 – 1.017) | 0.32 |
|  |  |  |  |  |  |  |  |  |  |  |  |  |  |
| Respiratory | 0 | 0.978  (0.958 – 0.998) | 0.987  (0.966 –  1.008) | 0.982  (0.966 – 1.011) | 0.35 | 0.994  (0.975 – 1.014) | 0.999  (0.980 – 1.019) | 1.001  (0.988 – 1.015) | 0.73 | 0.996  (0.978 – 1.016) | 0.998  (0.980 – 1.016) | 0.997  (0.984 – 1.011) | 0.66 |
|  | 3-day | 0.975  (0.950 – 1.001) | 0.995  (0.968 –  1.022) | 0.993  (0.972 – 1.015) | 0.73 | 0.987  (0.961 – 1.013) | 0.995  (0.968 – 1.022) | 1.005  (0.985 – 1.026) | 0.40 | 0.990  (0.965 – 1.015) | 0.996  (0.971 – 1.022) | 0.998  (0.978 – 1.019) | 0.88 |

ORs reflect a 6.63 µg/m^3^ change in PM_2.5_. All models are adjusted for 3-day mean ambient temperature (cubic splines) and relative humidity and

daily counts of hospitalization for influenza (in respiratory mortality models only).

**Figure S1**. Concentration-response curves using natural cubic splines with 3 degrees of freedom for the associations between exposure to 3-day mean PM_2.5_ and nonaccidental, cardiovascular and respiratory mortality in 24 cities across Canada (1998 – 2011). Solid lines reflect odds ratios and dashed lines reflect 95% confidence intervals.

**
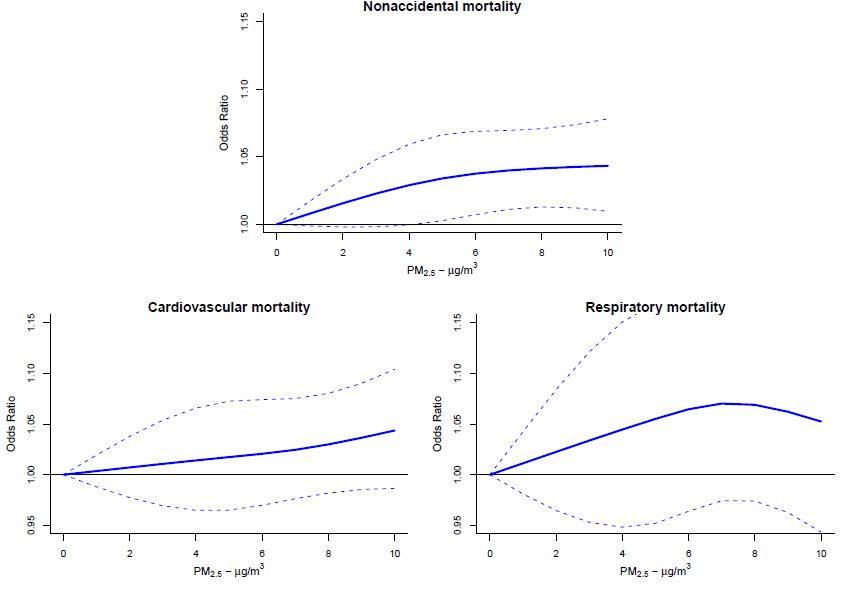
**

All models are adjusted for 3-day mean ambient temperature (cubic splines) and relative humidity and daily counts of hospitalization for influenza (in respiratory mortality models only).

**Figure S2**. Concentration-response curves using natural cubic splines with 3 degrees of freedom for the association between exposure to 3-day mean O_x_ and nonaccidental, cardiovascular and respiratory mortality in 24 cities across Canada (1998 – 2011). Solid blue line reflects odds ratios and gray area reflect 95% confidence intervals.


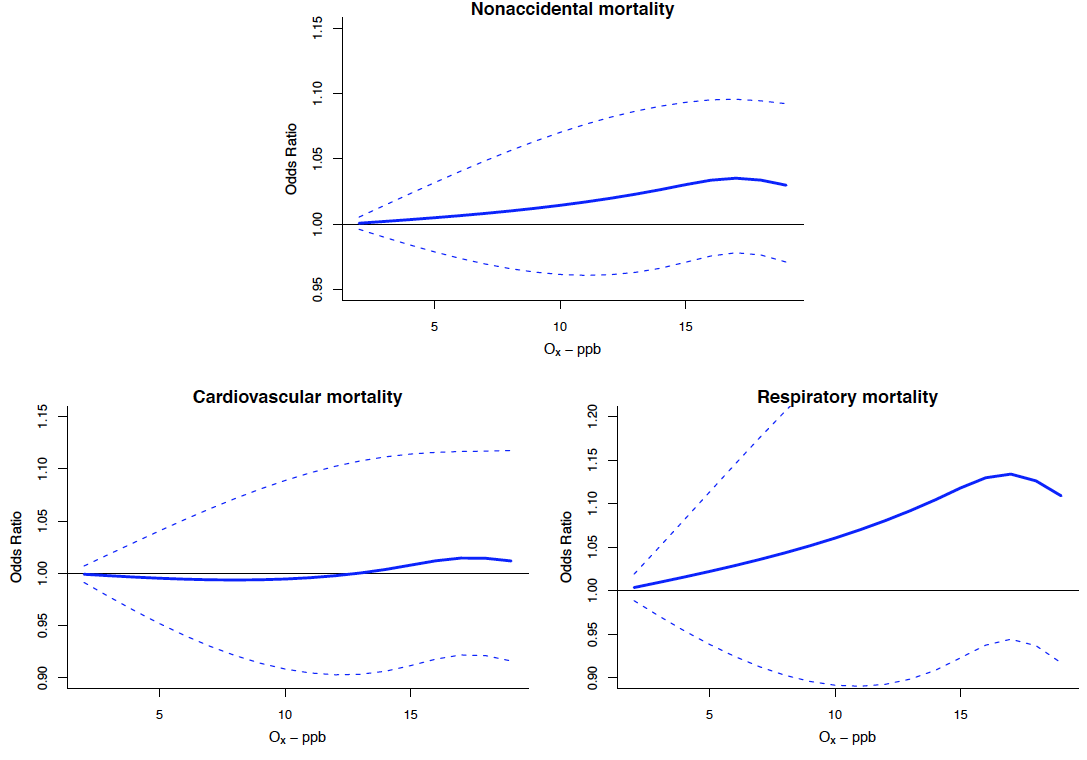


All models are adjusted for 3-day mean ambient temperature (cubic splines) and relative humidity and daily counts of hospitalization for influenza (in respiratory mortality models only).
